# Variation of Finger Activation Patterns Post-stroke Through Non-invasive Nerve Stimulation

**DOI:** 10.3389/fneur.2018.01101

**Published:** 2018-12-13

**Authors:** Henry Shin, Yang Zheng, Xiaogang Hu

**Affiliations:** Joint Department of Biomedical Engineering, University of North Carolina at Chapel Hill and North Carolina State University, Raleigh, NC, United States

**Keywords:** proximal nerve stimulation, neuromuscular electrical stimulation, stroke, electromyography, finger flexion

## Abstract

**Purpose:** A transcutaneous proximal nerve stimulation technique utilizing an electrode grid along the nerve bundles has previously shown flexible activation of multiple fingers. This case study aimed to further demonstrate the ability of this novel stimulation technique to induce various finger grasp patterns in a stroke survivor.

**Methods:** An individual with chronic hemiplegia and severe hand impairment was recruited. Electrical stimulation was delivered to different pairs of an electrode grid along the ulnar and median nerves to selectively activate different finger flexor muscles, with an automated electrode switching method. The resultant individual isometric flexion forces and forearm flexor high-density electromyography (HDEMG) were acquired to evaluate the finger activation patterns. A medium and low level of overall activation were chosen to gauge the available finger patterns for both the contralateral and paretic hands. All the flexion forces were then clustered to categorize the different types of grasp patterns.

**Results:** Both the contralateral and paretic sides demonstrated various force clusters including single and multi-finger activation patterns. The contralateral hand showed finger activation patterns mainly centered on median nerve activation of the index, middle, and ring fingers. The paretic hand exhibited fewer total activation patterns, but still showed activation of all four fingers in some combination.

**Conclusion:** Our results show that electrical stimulation at multiple positions along the proximal nerve bundles can elicit a select variety of finger activation patterns even in a stroke survivor with minimal hand function. This system could be further implemented for better rehabilitative training to help induce functional grasp patterns or to help regain muscle mass.

## Introduction

Following a stroke, a majority of individuals have paresis due to a loss of excitatory input and subsequent complications, such as disuse atrophy (1) and altered spinal organization (2–4). This loss of voluntary control of muscle activation often limits activities of daily living. Neuromuscular electrical stimulation (NMES) has been widely utilized both in the clinic and in research settings to help restore atrophied muscle and lost functions (5–7). Electrical stimulation has been particularly successful with post-stroke survivors for functional recovery (8–10). Research in NMES also aims to restore functional activation of muscles, such as the restoration of hand grasps (11).

Traditionally, NMES uses large electrode pads, targeting the distal branches of the nerve, known as the motor point stimulation (12). Although stimulation of the motor point is straightforward methodologically, NMES is limited to localized muscle activation, which limits its functional efficacy and also leads to rapid muscle fatigue (13). Advances in NMES techniques to alleviate these issues involve various multi-electrode techniques, which can stimulate multiple small regions of the muscle to help distribute the current and potentially activate more muscle fibers (14, 15). Crema et al. has also demonstrated flexible activation of multiple fingers using a multi-electrode array across the forearm and hand (16). Other approaches to NMES involve stimulation of the nerve bundle prior to branching and innervating a muscle, which has shown to allow for a larger area of muscle activation and potentially reduce long-term fatigue effects (17–19).

Recent developments have demonstrated the capabilities of an alternative non-invasive transcutaneous electrical nerve stimulation method targeting the ulnar and median nerves proximal to the elbow to flexibly activate individual and multiple fingers (20, 21). In addition, this technique shows the ability to delay the force decline (22, 23). A stimulation electrode grid placed along the two nerves allows us to activate different muscles or muscle portions to elicit varied desired movements, but manually switching between different electrode pairs is time-consuming. To shorten this process, an automated electrode pair searching method has been developed and tested on intact control subjects (24). This new method can further categorize the total available sets of finger activation patterns across the entire electrode grid, providing valuable information on electrode selection and the force generation capacity of stroke muscles. However, the efficiency of this method has not been tested on stroke survivors. Therefore, this case study recruited a control subject and a stroke survivor with severe weakness of the right arm, and evaluated the available finger activation patterns of the subjects. Our results showed varied activation of multiple fingers from both subjects. Further development of this stimulation technique can provide valuable alternatives to current rehabilitation for the restoration of hand movements.

## Methods

### Case Report

A 54-year-old male who had a left hemisphere ischemic stroke 2 years ago was recruited. The participant had limited voluntary motion in the arm and hand with significant muscle atrophy but had no cognitive impairments. The average ratio of the subject's maximum finger forces between hands was 0.076, and the subject's Chedoke-McMaster Stroke Assessment hand score was 2, both indicating severe impairment. A 35-year-old male participant was also recruited as a neurologically-intact control subject for comparison. This study was carried out in accordance with the recommendations of the Institutional Review Board (IRB) of the University of North Carolina at Chapel Hill with written informed consent from the subject. The subject gave written informed consent in accordance with the Declaration of Helsinki. The protocol was approved by the local IRB. Additional written informed consent was obtained from the subject for the publication of this case report.

### Experimental Setup

**Figure 1:**
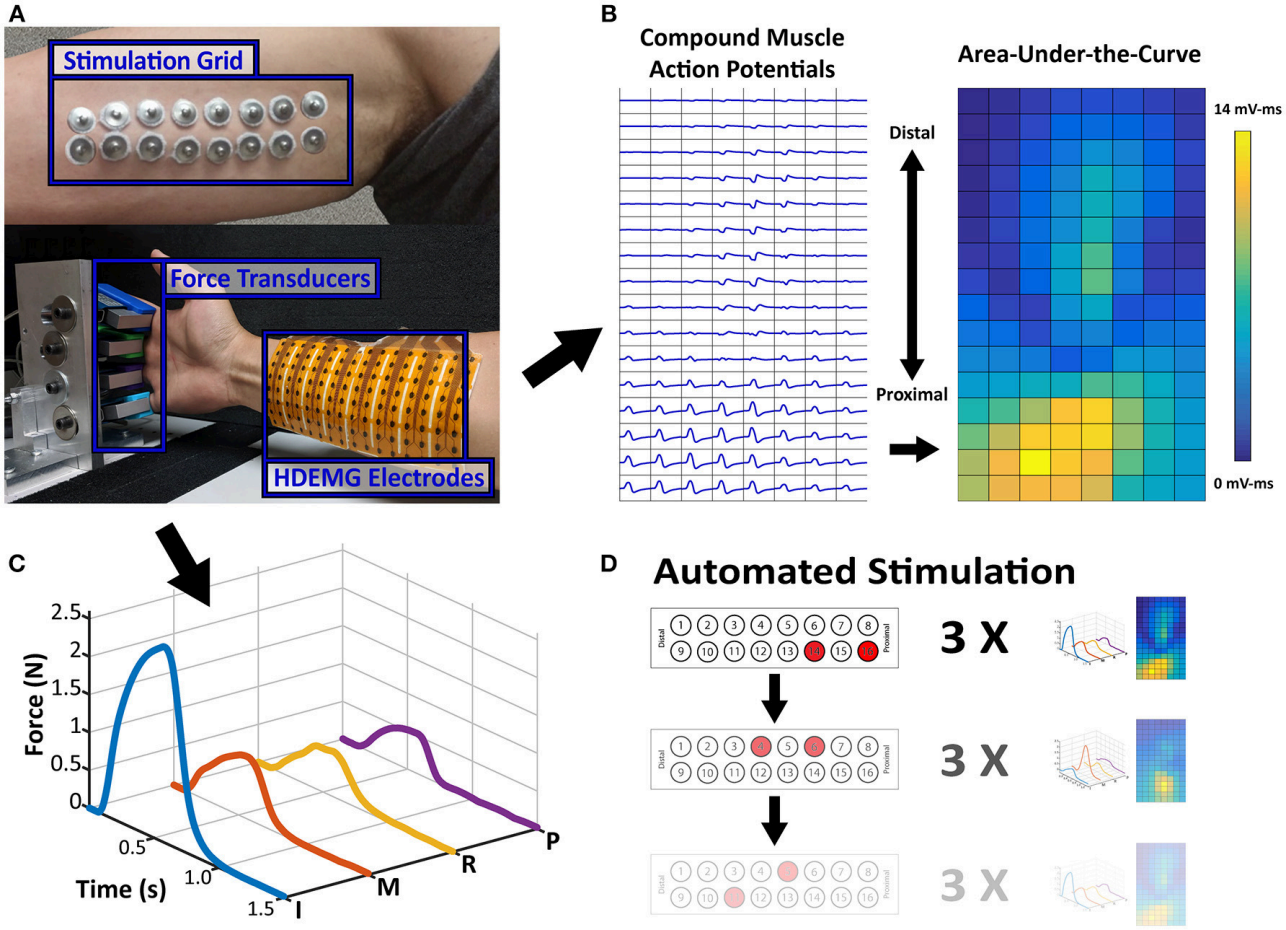
Experimental Setup and Data Samples. **(A)** Stimulation Electrode Array and Force/HDEMG Setup. Processed Data samples are displayed adjacent to the setup figure. **(B)** The EMG map is the spatial map of calculated AUC values from each EMG channel's CMAP and **(C)** the Force Profile is the smoothed force of each finger. **(D)** Sample Depiction of Automated Stimulation Procedure. Each stimulation pair can be paired with an EMG activity map and a force profile, which is the repetition of 3 stimulations.

To compare the proximal nerve stimulation method, the stroke subject's paretic and contralateral sides were tested on two separate occasions. The control subject was tested on the dominant arm. For each experiment, a 2 × 8 stimulation electrode grid was placed along the medial side of the upper arm below the biceps muscle where the ulnar and median nerves are more superficial (Figure 1A). All 16 stimulation electrodes were individually connected to a switch matrix (34904A, Agilent Technologies), which could be programmatically controlled. The switch matrix was then connected to a multi-channel stimulator (STG4008, Multichannel Systems), which could also be digitally controlled to deliver any range of current amplitudes between 0 and 16 mA, with a resolution of 20 μs. A brief cycle of 200 μs pulse width, 4 mA amplitude, and 30 Hz stimulation was delivered to every electrode to identify notably uncomfortable electrode combinations, which were then disabled. Following the stimulation setup, the skin of the anterior forearm was cleaned to reduce skin impedance for recording high density electromyography (HDEMG). An 8 × 16 HDEMG array (OT Bioelettronica)with a 10 mm interelectrode distance, was placed over the flexor compartment of the forearm (Figure 1B), and the 128 EMG channels were band-pass filtered at 10–900 Hz, with a gain of 500, and sampled at 5,120 Hz (EMG-USB2+, OT Bioelettronica). Lastly, each of the four fingers was individually secured to a uni-axial force transducer (SM-200N, Interface Inc.). Each finger was secured just above the metacarpophalangeal (MCP) joint (Figure 1A). The rest of the wrist and thumb were restricted to minimize force contamination. The force was recorded at 1,000 Hz. All the data recording and stimulation control were unified in a custom MATLAB GUI (Mathworks).

### Automated Stimulation Procedure

Once the setup was completed, the subjects were asked to perform maximum voluntary contractions (MVC) with each of the fingers individually and all 4 together. The stimulation procedure was composed of four steps:

#### Initial Medium-Level Grid Stimulation

An initial current level was chosen which can elicit some noticeable finger force without excessive contraction. For the paretic and contralateral sides of the stroke subject, the current levels were 6.5 and 4.5 mA, respectively, and 5 mA for the control subject. At this initial current level, all the different pair permutations were automatically switched and stimulated to test all the stimulation locations (120 maximum pairs). The bipolar stimulation consisted of trains of matched biphasic 200 μs pulse width and 30 Hz pulses. The stimulation was active for 0.5 s, and at rest for 1 s, while both the force and EMG were simultaneously recorded. Each pair was repeated 3 times before the stimulation was switched to the next pair (Figure 1D).

#### Max Force Selection and Activation Range Estimation

Once all available electrode pairs were stimulated, the subject was given a minute of rest while the GUI identified the stimulation electrode pair which resulted in the strongest average force across all the fingers within a single repetition. This electrode pair was then used to estimate the current-force relation across all of the other electrode pairs. Stimulation at 1 mA intervals was conducted to determine a rough estimate of the minimum and medium-high activation levels (relative to the current of the initial search) needed for each tested hand.

#### Current-Force Relation

Randomly chosen levels of current between these ranges (Paretic: 2–8 mA, Contralateral: 2–5 mA, Control: 2–7 mA) were stimulated using the same previous parameters, but with an increased 2-s rest between successive stimulations to reduce possible fatigue. Only the force was recorded with each stimulation, and the peak averaged force was calculated. The resultant Current-Force curve was then normalized to the corresponding averaged MVC.

#### Low-Level Grid Stimulation

The current value which activated around 5% MVC was selected to represent a level where low levels of finger motions were available. As the paretic MVC was already low, a value was chosen which was close to the lower take-off region of the current-force relation. The chosen values for the paretic and contralateral sides were 6 and 4.3 mA, respectively. The low-level selected for the control subject was 4 mA. The entire electrode grid underwent the automated stimulation procedure at these new current levels.

### Data Analysis

The data were processed to simplify its comparison across electrode pairs. First the 30 ms of HDEMG data after each stimulation pulse were aligned and averaged to form a single compound muscle action potential (CMAP), which was again averaged across the 3 repetitions for each electrode pair. The Area-Under-the-Curve (AUC) of each CMAP was calculated as a measure of overall activity of a single EMG channel. These AUC Values were then placed in a 2D array which corresponded to its physical location on the forearm, and this overall heat map was used to compare the muscle activity. Additionally, the force data were smoothed using a 100-ms window with 1-ms steps and averaged across the 3 repetitions. Examples of the processed HDEMG and force data are shown in Figures 1B,C, respectively. Any electrode pairs which did not produce at least 0.1N of force in any single finger were excluded from further analysis.

Hierarchical clustering was utilized to categorize the different grasp patterns based on the force data. Since the force data was retained as a 1,500 × 4 array, a 2D correlation coefficient was calculated between the averaged force data of different electrode pairs. This value was considered as the distance between two electrode pairs, and then the complement of this correlation distance (1–Corr. Coef.), also known as the dissimilarity, was calculated between stimulation locations. Using an inconsistency cutoff of 1.1, the initial hierarchical clusters were then used as a starting point to further refine the force patterns caused by each stimulation location. The silhouette coefficient was used as a measure of cluster validity, and therefore each member of each group was shuffled until the average silhouette coefficient of each group was maximized. For each resultant final cluster group, the average ratio of force between each finger was used to threshold whether or not the finger was active. Each of these clusters were therefore labeled by its finger activation.

Lastly, the EMG Activity AUC Maps of each force cluster were correlated with each other to quantify the similarity between the EMG activation of each force cluster. A high correlation indicates that electrode pairs within a force cluster also produces similar EMG activity, and inversely, a low correlation indicates that the electrode pairs within a cluster may produce similar force, but through different muscle portions.

### Results

The maximum voluntary forces obtained from the subject showed a large disparity between sides. On the contralateral side, the subject's individual finger forces were 19.9, 21.1, 30.1, and 21.9 N for the Index, Middle, Ring, and Pinky fingers, respectively (Average: 23.2 N). For the paretic side, the finger forces were 0.7, 3.6, 2.4, and 0.3 N (Average: 1.8 N). The finger maximums for the control subject were 26.7, 26.3, 20.6, and 26.9 N (Average: 25.1 N). These values were in a similar range as the stroke subject's contralateral side. Although initially obtained to normalize the elicited forces, due to the very low forces of the paretic side, the absolute forces were reported.

**Figure 2:**
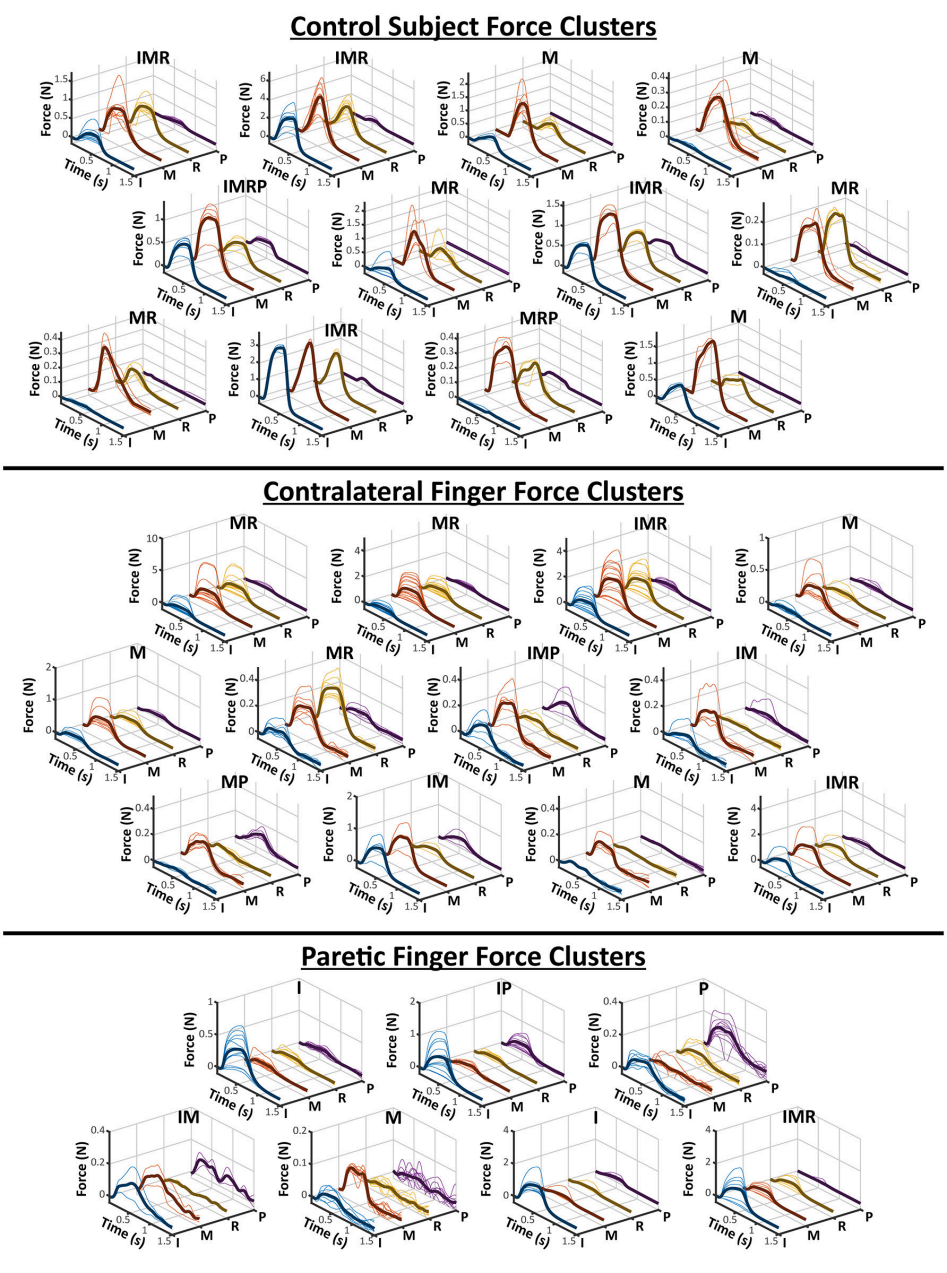
All Finger Force Clusters. The hierarchical clustering results of a control subject and the two sides of a stroke subject is visualized here. As shown in Figure 1, each finger is individually plotted from the force profiles of each electrode pair in a cluster. The average of all of the force profiles in a cluster are also shown as the darker solid line within each axes. The labels above each cluster represent which fingers were deemed “active” based on its relative ratio to the other fingers. The letters for each finger are, I-Index, M-Middle, R-Ring, and P-Pinky.

The clusters obtained from the contralateral and paretic hands, as well as the control subject, are shown in Figure 2. The labels on the top of each cluster indicates which of the four recorded fingers were active. The Contralateral hand and the control subject showed a variety of single and multi-finger activation patterns which were mostly an activation of the Index, Middle, and Ring fingers, but also a few Pinky. Similarly, the Paretic hand also resulted in several clusters of activation patterns, although fewer than the contralateral side. The paretic hand resulted in relatively more clusters with only a single finger being active, but still had a few two and three finger activations. Overall, the contralateral hand and the control subject clusters show that the electrode grid had strong preference to the median nerve (Index-Middle-Ring), whereas the paretic-side electrode grid may have had a more evenly divided placement between the two desired nerve bundles.

**Figure 3:**
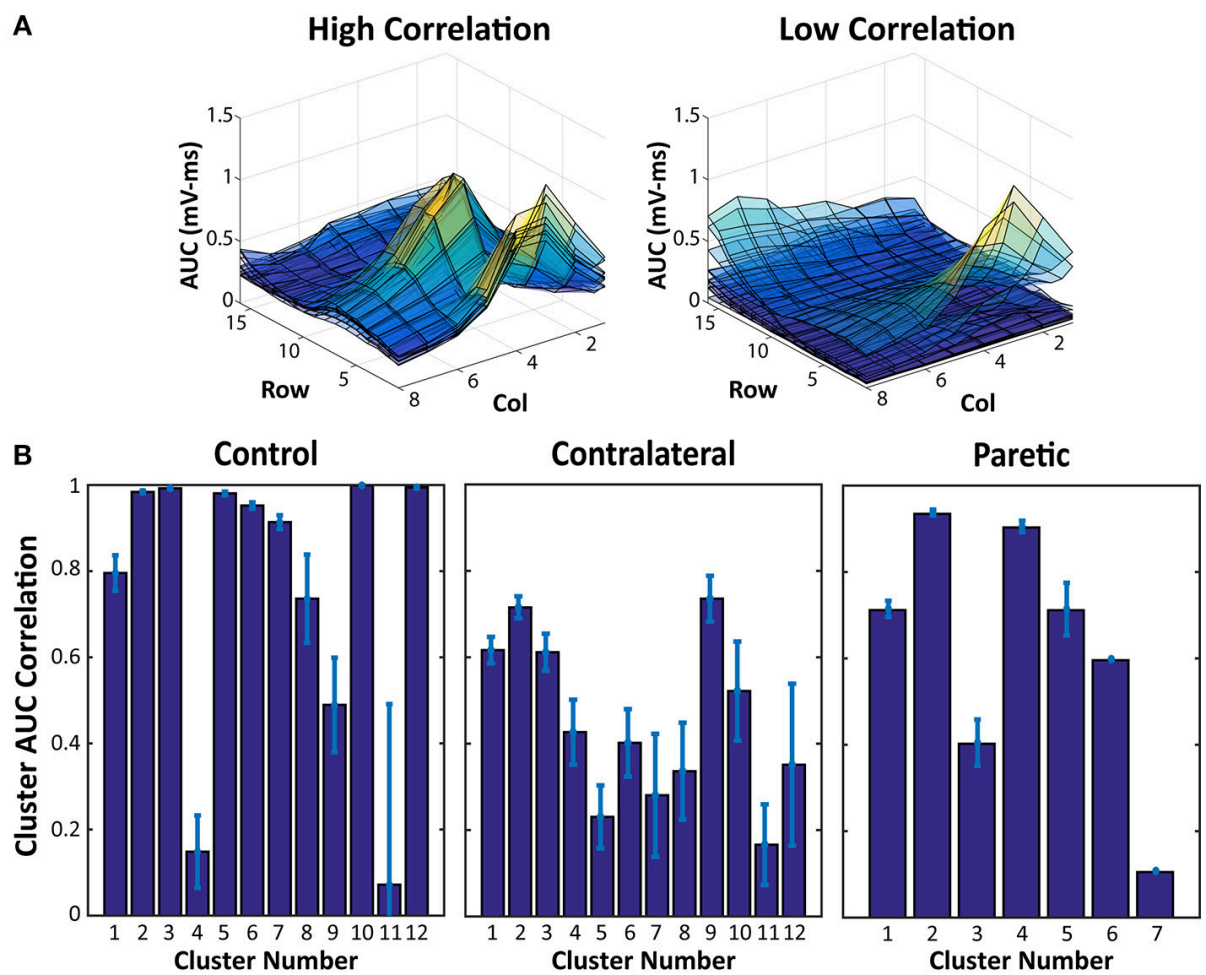
EMG AUC Map Correlation Examples and Cluster Averages. **(A)** Two samples of overlaid EMG AUC values from all of the electrode pairs within a force cluster. High Correlation indicates EMG activity within a cluster with very similar activation patterns, whereas the lower correlation values are a result of more varied EMG AUC Maps. **(B)** EMG AUC Correlation values from each cluster in Figure 2. Average and Standard Error are shown.

The results of the AUC Correlation analysis are shown in Figure 3. Figure 3A shows two examples of EMG activity with high cluster correlation and low cluster correlation. Figure 3B illustrates the individual cluster AUC Correlation for two sides of the stroke subject and the control subject. These results suggest that for each force cluster in Figure 2, there is a high variability of EMG correlation. Some force clusters also have high EMG activity correlation, whereas other force clusters may have more varied EMG activity, and therefore lower correlation.

## Discussion

In the current case report, an individual with chronic stroke associated muscle weakness was tested with our proximal nerve stimulation system alongside a neurologically-intact control subject to evaluate the capabilities to elicit specific finger activations in highly paretic muscle. Overall, our results demonstrate that our stimulation system is able to activate various different fingers on both the contralateral and paretic sides of this subject.

As a survey of the available finger activations of the stimulation method, our results highlight a few important aspects of the activated finger patterns. Similar to previous results (24), a majority of the finger activations were those of the Index, Middle, and Ring fingers. These correspond to the median nerve, and therefore it can be concluded that the placement of the electrode stimulation array was preferential to the median nerve, especially for the contralateral hand and in the control subject. The sets of force clusters from these two conditions also demonstrate similar ranges of single and multi-finger activation patterns. As for the paretic hand, the activation of the Pinky finger suggests that more of the ulnar nerve was also accessible. As the paretic biceps muscle was also visibly atrophied when the stimulation electrodes were applied, the underlying nerve bundles may have been more easily accessible. Additionally, the results suggest that different electrode pairs are able to activate different portions of the corresponding nerves. Although the different clusters are a *post-hoc* attempt at organizing the finger forces generated by each electrode pair, in reality, each elicitable finger pattern lies along a continuum of finger activation levels. Different electrode pairs impose a unique electrical field onto the nerve, and thus activates a unique subset of motor and/or sensory axons. As shown in the contralateral force clusters, many of these subset axons project to muscles spanning multiple fingers, but a small number of the electrodes can partially activate a single finger. The different clusters help to identify which sets of electrode pairs can elicit desirable finger grasp patterns. Additionally, although anatomical landmarks were used during the stimulation grid placement, small variations in the location of the electrodes could inevitably lead to different sets of activation even within the same subject. Therefore, although there may be inter-session differences in the exact number and range of force clusters, the general similarity between the control subject and the contralateral side suggest that the stimulation grid is able to activate similar patterns of finger movement.

Although the obtained number of clusters are not necessarily indicative of any physiological correlation with muscle function, it is important to note that the paretic side does have notably fewer number and variety of force clusters (12 on contralateral/control vs. 7 on paretic side). This may be due to several factors that occur in paretic muscle after stroke. The first is due to the significant atrophy and weakness of the right arm and hand seen in the participant. As the overall stimulated force level was still very low, the limited muscle mass may not have been able to generate observable levels of force in some activation patterns. Alternatively, various losses in the motor unit (MU) numbers as well as reinnervation of existing MUs are also common occurrences after stroke (25, 26). This may alter the various subsets of activation available through nerve stimulation. Further studies are needed to confirm these possibilities, as the lower number of clusters may also simply be due to the chance involved with electrode placement. Clearly, additional testing involving a large stroke cohort is necessary.

Along with the overview of the different force clusters, as an estimation of the total available activation patterns, the EMG AUC Map correlation within each cluster also provides further insight into the actual EMG activity from each stimulation. Figure 3 shows that within each force cluster, there may be a wide distribution of similar and dissimilar EMG activity. A force cluster that has high EMG AUC correlation, may imply a set of electrode pairs which consistently activate the same portion of muscles. Contrastingly, a force cluster that has a low EMG AUC correlation may suggest that the set of electrode pairs in the cluster is able to activate different portions of muscles even with the same resultant physical activity. This feature may have far reaching effects regarding prolonging use with stimulation redundancy. Various groups have shown that, distributed muscle activation using multiple pads is able to lead to reduced fatigability in NMES (27–29). Being able to alternate between different electrode pairs which recruit different muscles with similar functional outputs could reduce muscle fatigue in the stimulation. Further investigation on the relation between the force variability and EMG variability is necessary to better understand the different electrode pair choices and their impacts on force production.

## Concluding Remarks

The current study demonstrates the variety of finger activation patterns that are accessible through our proximal nerve stimulation method. Both the contralateral and paretic sides of a stroke subject were able to be successfully stimulated to produce a number of multi-finger movements along with a few isolated single fingers. The contralateral hand, in particular, was able to elicit a similar variety of finger activation patterns as seen in the control subject. Further development of the technique can also be combined with radial nerve stimulation to also include hand opening, which is just as important for stroke survivors. The automated electrode searching with the force clustering can help rapidly identify the feasible sets of electrode pairs, which can allow us to develop an auto-calibration method between sessions/days, which can then be applied to any future uses for rehabilitation.

## Author Contributions

HS and XH conceptualized the study design. HS and YZ performed the experiment. HS analyzed the data and drafted the manuscript. HS, YZ, and XH revised the manuscript and approved the final version of the manuscript.

### Conflict of Interest Statement

The authors declare that the research was conducted in the absence of any commercial or financial relationships that could be construed as a potential conflict of interest.
